# Supplementary material for: Genetic Diversity, Analysis of Some Agro-Morphological and Quality Traits, and Utilization of Plant Resources of Coriander (Coriandrum sativum) Supported with Cluster and Multivariate Analyses
Source: Biology (Basel). 2024 Oct 24;13(11):866. doi: 10.3390/biology13110866 (PMC11592299; doi:10.3390/biology13110866)
Supplement: Supplementary file 1 [file biology-13-00866-s001.zip › biology-3227131-supplementary.pdf]

Table S1. 50% seedling, flowering and fruit setting days of the different coriander genotypes.

G/C	50% SD	G/C	50% SD	G/C	50% FD	G/C	50% FD	G/C	50% FSD	G/C	50% FSD
PI 669966	34.57C-K	Ames 23634	34.57C-K	PI 669966	56.20d-m	Ames 23634	56.20d-m	PI 669966	66.00b-g	Ames 23634	69.00U-g
PI 669965	33.57E-M	Ames 23632	35.57C-I	PI 669965	56.20d-m	Ames 23632	56.60b-l	PI 669965	66.00b-g	Ames 23632	69.00U-g
PI 669964	34.57C-K	Ames 23627	33.57E-M	PI 669964	58.20V-ı	Ames 23627	70.60ABC	PI 669964	66.00b-g	Ames 23627	67.40Z-g
PI 669963	35.57C-I	Ames 23626	31.57J-O	PI 669963	58.20V-ı	Ames 23626	56.60b-l	PI 669963	67.00Z-g	Ames 23626	65.40c-g
PI 669962	35.57C-I	Ames 23625	32.57H-O	PI 669962	57.20a-k	Ames 23625	67.60B-I	PI 669962	67.00Z-g	Ames 23625	67.40Z-g
PI 669961	35.57C-I	Ames 23624	25.57S	PI 669961	56.20d-m	Ames 23624	69.60A-E	PI 669961	67.00Z-g	Ames 23624	85.40C-H
PI 669960	34.57C-K	Ames 23623	31.57J-O	PI 669960	56.20d-m	Ames 23623	54.60h-m	PI 669960	67.00Z-g	Ames 23623	79.40F-Q
PI 669959	34.57C-K	Ames 23622	34.57C-K	PI 669959	59.20S-e	Ames 23622	56.60b-l	PI 669959	67.00Z-g	Ames 23622	68.40V-g
PI 664512	33.57E-M	Ames 23621	33.57E-M	PI 664512	64.20I-P	Ames 23621	54.60h-m	PI 664512	79.00F-R	Ames 23621	80.40F-P
PI 633685	36.57B-F	Ames 23620	32.57H-O	PI 633685	68.20B-H	Ames 23620	54.60h-m	PI 633685	80.00F-Q	Ames 23620	69.40T-f
PI 531296	33.57E-M	Ames 23619	32.57H-O	PI 531296	70.20A-D	Ames 23619	54.60h-m	PI 531296	79.00F-R	Ames 23619	67.40Z-g
PI 531293	35.57C-I	Ames 23618	29.57N-Q	PI 531293	68.20B-H	Ames 23618	68.60A-G	PI 531293	79.00F-R	Ames 23618	65.40c-g
PI 502320	37.57A-D	Ames 23616	31.57J-O	PI 502320	60.20Q-c	Ames 23616	55.60e-m	PI 502320	86.00C-G	Ames 23616	80.40F-P
PI 483232	36.57B-F	Ames 23614	31.57J-O	PI 483232	59.20S-e	Ames 23614	63.60J-Q	PI 483232	81.00E-O	Ames 23614	68.40V-g
PI 478378	35.57C-I	Ames 21655	31.57J-O	PI 478378	55.20g-m	Ames 21655	55.60e-m	PI 478378	73.00P-c	Ames 21655	84.40D-I
PI 274290	33.57E-M	Ames 21108	32.57H-O	PI 274290	62.20L-U	Ames 21108	55.60e-m	PI 274290	65.00d-g	Ames 21108	66.40a-g
PI 269472	33.57E-M	Ames 21105	32.57H-O	PI 269472	68.20B-H	Ames 21105	68.60A-G	PI 269472	83.00E-L	Ames 21105	66.40a-g
PI 269470	32.57H-O	Ames 20048	31.57J-O	PI 269470	56.60b-l	Ames 20048	69.60A-E	PI 269470	78.00H-S	Ames 20048	83.40E-K
PI 268378	33.57E-M	Ames 20046	30.37L-P	PI 268378	57.60Y-j	Ames 20046	70.60ABC	PI 268378	74.20M-a	Ames 20046	86.40B-F
PI 256061	33.57E-M	Ames 19089	30.37L-P	PI 256061	56.60b-l	Ames 19089	53.00lmn	PI 256061	79.20F-Q	Ames 19089	88.40A-E
PI 253146	32.57H-O	Ames 18596	37.37A-D	PI 253146	55.60e-m	Ames 18596	69.00A-G	PI 253146	74.20M-a	Ames 18596	67.00Z-g
PI 249115	37.57A-D	Ames 18595	29.37OPQ	PI 249115	65.60F-M	Ames 18595	69.00A-G	PI 249115	70.20S-f	Ames 18595	81.00E-O
PI 196843	35.57C-I	Ames 18594	31.37J-O	PI 196843	59.60R-d	Ames 18594	69.00A-G	PI 196843	92.20A-D	Ames 18594	78.00H-S
PI 193770	34.57C-K	Ames 18592	36.37B-G	PI 193770	58.60T-g	Ames 18592	63.00K-S	PI 193770	72.20Q-d	Ames 18592	80.00F-Q
PI 193769	37.57A-D	Ames 18591	37.37A-D	PI 193769	66.60D-K	Ames 18591	62.00M-V	PI 193769	71.20R-e	Ames 18591	77.00I-T
PI 193493	34.57C-K	Ames 18590	39.37AB	PI 193493	67.60B-I	Ames 18590	68.00B-I	PI 193493	93.20ABC	Ames 18590	80.00F-Q
PI 174129	36.57B-F	Ames 18589	37.37A-D	PI 174129	67.60B-I	Ames 18589	63.00K-S	PI 174129	84.20E-J	Ames 18589	81.00E-O
PI 172808	37.57A-D	Ames 18588	29.37O-R	PI 172808	58.60T-g	Ames 18588	54.00j-m	PI 172808	94.20AB	Ames 18588	77.00I-T
PI 171592	34.57C-K	Ames 18585	30.37L-P	PI 171592	68.60A-G	Ames 18585	67.00C-J	PI 171592	71.20R-e	Ames 18585	67.00Z-g
PI 170320	34.57C-K	Ames 18587	33.37E-M	PI 170320	69.60A-E	Ames 18587	66.00E-L	PI 170320	81.20E-N	Ames 18587	80.00F-Q

PI 170319	33.57E-M	Ames 18583	33.37E-M	PI 170319	60.60O-a	Ames 18583	62.00M-V	PI 170319	96.20A	Ames 18583	83.00E-L
Pelmus	33.33F-M	Ames 18582	36.37B-G	Pelmus	59.00T-g	Ames 18582	68.00B-I	Pelmus	78.50G-R	Ames 18582	76.00K-V
Gürbüz	33.17F-N	Ames 18581	33.37E-M	Gürbüz	59.17S-f	Ames 18581	61.00O-a	Gürbüz	73.17O-c	Ames 18581	77.00I-T
Gamze	36.57B-F	Ames 18580	29.37OPQ	Gamze	61.50N-V	Ames 18580	67.00C-J	Gamze	75.67K-Y	Ames 18580	78.00H-S
Erbaa	36.57B-F	Ames 18578	36.37B-G	Erbaa	55.33f-m	Ames 18578	67.00C-J	Erbaa	78.50G-R	Ames 18578	81.00E-O
Arslan	36.57B-F	Ames 18577	33.37E-M	Arslan	56.20d-m	Ames 18577	62.00M-V	Arslan	67.17Z-g	Ames 18577	81.00E-O
Ames 4998	32.97F-O	Ames 18575	29.37OPQ	Ames 4998	60.40P-b	Ames 18575	68.00B-I	Ames 4998	76.40J-U	Ames 18575	82.00E-M
Ames 23640	31.97I-O	Ames 18574	24.37S	Ames 23640	55.20g-m	Ames 18574	61.00O-a	Ames 23640	69.00U-g	Ames 18574	78.00H-S
Ames 29174	32.57H-O	Ames 18573	29.97M-P	Ames 29174	68.60A-G	Ames 18573	53.40klm	Ames 29174	81.20E-N	Ames 18573	73.00P-c
Ames 29173	32.57H-O	Ames 18572	35.97B-H	Ames 29173	69.60A-E	Ames 18572	53.40klm	Ames 29173	84.20E-J	Ames 18572	62.40fg
Ames 29172	30.97K-O	Ames 18571	36.97B-E	Ames 29172	55.60e-m	Ames 18571	62.40L-T	Ames 29172	94.20AB	Ames 18571	62.40fgh
Ames 27870	32.97F-O	Ames 18570	26.97P-S	Ames 27870	55.60e-m	Ames 18570	52.40mn	Ames 27870	70.20S-f	Ames 18570	73.40N-b
Ames 27772	33.97D-L	Ames 18569	25.97QRS	Ames 27772	55.20g-m	Ames 18569	69.40A-F	Ames 27772	69.20T-g	Ames 18569	62.40fg
Ames 27771	32.97F-O	Ames 18568	24.97S	Ames 27771	55.20g-m	Ames 18568	53.40k-n	Ames 27771	68.00Y-g	Ames 18568	83.40E-K
Ames 27770	32.97F-O	Ames 18567	35.97B-H	Ames 27770	67.20C-J	Ames 18567	61.40N-Y	Ames 27770	68.00Y-g	Ames 18567	61.40gh
Ames 27392	31.97I-O	Ames 18566	33.97D-L	Ames 27392	65.20G-N	Ames 18566	64.40H-O	Ames 27392	83.00E-L	Ames 18566	82.40E-L
Ames 27391	32.97F-O	Ames 18565	32.97F-O	Ames 27391	69.20A-F	Ames 18565	68.40A-G	Ames 27391	83.00E-L	Ames 18565	81.40E-M
Ames 25170	33.97D-L	Ames 18564	31.97I-O	Ames 25170	61.20O-Z	Ames 18564	66.40D-K	Ames 25170	84.00E-J	Ames 18564	81.40E-M
Ames 24926	31.97I-O	Ames 18563	31.97I-O	Ames 24926	64.20I-P	Ames 18563	52.40mn	Ames 24926	81.00E-O	Ames 18563	82.40E-L
Ames 24923	33.97D-L	Ames 18561	34.97C-J	Ames 24923	69.20A-F	Ames 18561	57.40Z-j	Ames 24923	83.00E-L	Ames 18561	61.40gh
Ames 24921	33.97D-L	Ames 18559	37.97ABC	Ames 24921	58.20V-ı	Ames 18559	57.40Z-j	Ames 24921	81.00E-O	Ames 18559	74.40M-Z
Ames 24917	34.97C-J	Ames 18560	40.97A	Ames 24917	71.20AB	Ames 18560	58.40U-h	Ames 24917	70.00T-f	Ames 18560	65.40c-g
Ames 24909	33.97D-L	Ames 14363	36.97B-E	Ames 24909	72.20A	Ames 14363	56.40c-l	Ames 24909	95.00A	Ames 14363	65.40c-g
Ames 24907	31.97I-O	Ames 13900	35.97B-H	Ames 24907	58.20V-ı	Ames 13900	63.40J-R	Ames 24907	93.00ABC	Ames 13900	76.40J-U
Ames 23642	30.97K-O	Ames 13899	33.97D-L	Ames 23642	56.20d-m	Ames 13899	63.40J-R	Ames 23642	80.00F-Q	Ames 13899	75.40L-Y
Ames 23641	31.97I-O	Ames 12778	33.97D-L	Ames 23641	56.20d-m	Ames 12778	54.40ı-m	Ames 23641	69.00U-g	Ames 12778	62.40fg
Ames 23639	30.97K-O	Ames 10235	34.97C-J	Ames 23639	55.20g-m	Ames 10235	61.40N-Y	Ames 23639	73.00P-c	Ames 10235	76.40J-U
Ames 23635	34.57C-K	Ames 10234	33.97D-L	Ames 23635	55.20g-m	Ames 10234	53.4klm	Ames 23635	69.00U-g	Ames 10234	63.40efg

Differences between means indicated with the same letter are not significant. G/C: Genotypes/Cultivars, SD: Seedling days, FD: Flowering days, FSD: Fruit setting days.

Table S2. Plant height, branch number, umbel number and umbellet number values of different origin coriander genotypes.

G/C	PH	G/C	PH	G/C	BN	G/C	BN	G/C	UN	G/C	UN	G/C	UmbltN	G/C	UmbltN
PI 669966	32.68o-r	Ames 23634	56.16I-f	PI 669966	6.26U-l	Ames 23634	6.82P-j	PI 669966	15.07Q-l	Ames 23634	13.97R-n	PI 669966	43.38R-l	Ames 23634	39.79T-l
PI 669965	35.98l-r	Ames 23632	40.96e-r	PI 669965	6.26U-l	Ames 23632	5.02e-n	PI 669965	16.57P-h	Ames 23632	7.17h-q	PI 669965	53.03O-k	Ames 23632	12.59h-l
PI 669964	43.88a-q	Ames 23627	32.44o-r	PI 669964	6.46S-j	Ames 23627	4.02l-o	PI 669964	15.37Q-k	Ames 23627	7.65g-q	PI 669964	53.63O-k	Ames 23627	30.31Z-l
PI 669963	33.18n-r	Ames 23626	25.44rs	PI 669963	5.86Y-m	Ames 23626	2.82no	PI 669963	6.77j-q	Ames 23626	5.05m-q	PI 669963	26.23Z-l	Ames 23626	19.11e-l
PI 669962	38.38ı-r	Ames 23625	34.24m-r	PI 669962	8.46G-U	Ames 23625	3.62mno	PI 669962	12.57U-p	Ames 23625	7.05ı-q	PI 669962	44.83R-l	Ames 23625	26.51Z-l
PI 669961	30.18pqr	Ames 23624	67.24B-O	PI 669961	7.26N-e	Ames 23624	9.62C-M	PI 669961	12.37U-p	Ames 23624	11.85Y-q	PI 669961	47.43Q-l	Ames 23624	57.71N-ı
PI 669960	37.98j-r	Ames 23623	70.44A-M	PI 669960	6.66R-j	Ames 23623	11.62BC	PI 669960	8.37g-q	Ames 23623	15.45Q-k	PI 669960	28.43Z-l	Ames 23623	68.51L-d
PI 669959	40.38f-r	Ames 23622	46.64V-o	PI 669959	6.86P-ı	Ames 23622	5.82Y-m	PI 669959	18.97N-d	Ames 23622	9.85d-q	PI 669959	56.23O-j	Ames 23622	25.91a-l
PI 664512	71.78A-J	Ames 23621	73.84A-G	PI 664512	9.06E-P	Ames 23621	9.82C-L	PI 664512	32.77E-H	Ames 23621	12.45U-p	PI 664512	106.03E-N	Ames 23621	51.31P-k
PI 633685	73.18A-H	Ames 23620	47.84U-o	PI 633685	10.06C-K	Ames 23620	4.82f-n	PI 633685	28.97F-L	Ames 23620	5.65l-q	PI 633685	121.23D-J	Ames 23620	24.51b-l
PI 531296	55.78J-g	Ames 23619	55.04K-h	PI 531296	9.26D-N	Ames 23619	7.42M-d	PI 531296	21.77J-U	Ames 23619	8.85f-q	PI 531296	90.23G-S	Ames 23619	33.91Z-l
PI 531293	48.38T-o	Ames 23618	46.64V-o	PI 531293	6.26U-l	Ames 23618	6.02V-l	PI 531293	9.37e-q	Ames 23618	13.85R-n	PI 531293	42.63S-l	Ames 23618	39.71T-l
PI 502320	53.58N-j	Ames 23616	62.24D-V	PI 502320	8.86E-R	Ames 23616	8.62G-T	PI 502320	16.77O-g	Ames 23616	27.25F-N	PI 502320	75.43I-Z	Ames 23616	102.31F-O
PI 483232	46.78V-o	Ames 23614	50.84P-l	PI 483232	9.26D-N	Ames 23614	6.42S-k	PI 483232	22.77I-S	Ames 23614	11.05Z-q	PI 483232	94.83G-Q	Ames 23614	36.91V-l
PI 478378	52.38O-k	Ames 21655	83.24AB	PI 478378	7.66L-b	Ames 21655	11.02B-F	PI 478378	22.17J-T	Ames 21655	21.45J-V	PI 478378	75.03I-a	Ames 21655	92.51G-R
PI 274290	28.78qrs	Ames 21108	43.14b-q	PI 274290	6.46S-j	Ames 21108	4.82f-n	PI 274290	13.97R-n	Ames 21108	10.05c-q	PI 274290	43.23R-l	Ames 21108	40.51T-l
PI 269472	49.93Q-m	Ames 21105	40.34f-r	PI 269472	11.51BCD	Ames 21105	5.22d-m	PI 269472	20.82K-Y	Ames 21105	9.85d-q	PI 269472	90.38G-S	Ames 21105	38.71U-l
PI 269470	45.78Y-p	Ames 20048	64.24D-T	PI 269470	9.26D-N	Ames 20048	8.42G-U	PI 269470	19.97K-b	Ames 20048	32.05E-I	PI 269470	89.03H-T	Ames 20048	118.71D-K
PI 268378	42.10d-q	Ames 20046	76.24A-E	PI 268378	5.90Y-m	Ames 20046	10.42B-I	PI 268378	8.01g-q	Ames 20046	35.65DEF	PI 268378	36.31Y-l	Ames 20046	124.11D-I
PI 256061	63.30D-U	Ames 19089	73.64A-G	PI 256061	6.70Q-j	Ames 19089	9.22D-O	PI 256061	14.41R-m	Ames 19089	26.65F-N	PI 256061	63.51M-g	Ames 19089	138.91D-G
PI 253146	47.30U-o	Ames 18596	44.52Z-q	PI 253146	5.70a-m	Ames 18596	6.86P-ı	PI 253146	11.61Y-q	Ames 18596	4.37o-r	PI 253146	41.11S-l	Ames 18596	17.75f-l
PI 249115	39.60g-r	Ames 18595	72.32A-I	PI 249115	4.10k-n	Ames 18595	11.06B-F	PI 249115	12.01V-q	Ames 18595	24.17H-Q	PI 249115	42.91S-l	Ames 18595	148.55DEF
PI 196843	58.10G-d	Ames 18594	72.32A-I	PI 196843	7.90K-a	Ames 18594	9.06E-P	PI 196843	8.61g-q	Ames 18594	18.17N-f	PI 196843	50.71P-k	Ames 18594	67.35M-e
PI 193770	39.10h-r	Ames 18592	66.92C-P	PI 193770	4.10k-n	Ames 18592	7.46M-d	PI 193770	7.21h-q	Ames 18592	10.97a-q	PI 193770	42.31S-l	Ames 18592	44.15R-l
PI 193769	75.90A-F	Ames 18591	61.52D-Y	PI 193769	15.10A	Ames 18591	10.06C-K	PI 193769	52.41C	Ames 18591	19.77L-b	PI 193769	315.31B	Ames 18591	86.15H-V
PI 193493	52.90O-j	Ames 18590	54.52L-ı	PI 193493	8.85E-R	Ames 18590	8.86E-R	PI 193493	25.41G-P	Ames 18590	15.17Q-k	PI 193493	71.81K-c	Ames 18590	58.55N-ı
PI 174129	84.30A	Ames 18589	61.92D-Y	PI 174129	10.50B-H	Ames 18589	7.06N-g	PI 174129	19.81L-b	Ames 18589	6.57j-q	PI 174129	56.11O-j	Ames 18589	36.15Y-l
PI 172808	71.50A-J	Ames 18588	55.72J-g	PI 172808	5.30c-m	Ames 18588	7.06N-g	PI 172808	2.81qr	Ames 18588	7.97g-q	PI 172808	63.71M-g	Ames 18588	43.15S-l
PI 171592	49.10S-n	Ames 18585	59.12G-b	PI 171592	7.90K-a	Ames 18585	8.26H-V	PI 171592	11.81Y-q	Ames 18585	12.97T-p	PI 171592	75.11I-a	Ames 18585	40.35T-l
PI 170320	63.10D-U	Ames 18587	67.32B-O	PI 170320	6.90O-ı	Ames 18587	8.06J-Z	PI 170320	15.21Q-k	Ames 18587	14.17R-n	PI 170320	126.11D-H	Ames 18587	65.95M-f
PI 170319	49.90Q-m	Ames 18583	57.12H-e	PI 170319	4.70h-n	Ames 18583	6.66R-j	PI 170319	3.61pqr	Ames 18583	7.97g-q	PI 170319	60.51M-h	Ames 18583	27.95Z-l
Pelmus	58.52G-c	Ames 18582	57.72G-d	Pelmus	6.33T-l	Ames 18582	6.86P-ı	Pelmus	11.47Y-q	Ames 18582	8.77f-q	Pelmus	38.57V-l	Ames 18582	28.95Z-l
Gürbüz	59.37G-b	Ames 18581	73.92A-G	Gürbüz	7.50L-d	Ames 18581	10.66B-G	Gürbüz	12.90T-p	Ames 18581	31.97E-I	Gürbüz	53.43O-k	Ames 18581	118.35D-K
Gamze	65.63C-R	Ames 18580	69.32A-N	Gamze	7.63L-b	Ames 18580	8.86E-R	Gamze	18.87N-d	Ames 18580	18.97N-d	Gamze	68.20M-e	Ames 18580	72.75J-b
Erbaa	70.63A-L	Ames 18578	77.52A-D	Erbaa	7.47M-d	Ames 18578	9.66C-M	Erbaa	20.47K-Z	Ames 18578	29.37F-K	Erbaa	85.10H-Y	Ames 18578	108.35D-M
Arslan	49.45R-m	Ames 18577	64.72D-S	Arslan	7.17N-e	Ames 18577	9.66C-M	Arslan	11.97Y-q	Ames 18577	24.17H-Q	Arslan	40.27T-l	Ames 18577	87.95H-U
Ames 4998	50.00Q-m	Ames 18575	60.52E-Z	Ames 4998	5.74Z-m	Ames 18575	8.26H-V	Ames 4998	13.21T-o	Ames 18575	15.77Q-j	Ames 4998	41.79S-l	Ames 18575	75.35I-Z
Ames 23640	48.36T-o	Ames 18574	61.92D-Y	Ames 23640	7.22N-e	Ames 18574	7.66L-b	Ames 23640	10.77a-q	Ames 18574	11.37Y-q	Ames 23640	33.39Z-l	Ames 18574	58.55N-ı
Ames 29174	66.10C-Q	Ames 18573	66.72C-P	Ames 29174	7.90K-a	Ames 18573	10.06C-K	Ames 29174	28.61F-M	Ames 18573	26.17G-O	Ames 29174	85.31H-Y	Ames 18573	117.55D-L

Ames 29173	67.30B-O	Ames 18572	37.60j-r	Ames 29173	6.90O-ı	Ames 18572	5.74Z-m	Ames 29173	11.81Y-q	Ames 18572	6.21k-q	Ames 29173	75.11I-a	Ames 18572	0.19lm
Ames 29172	81.10ABC	Ames 18571	41.20e-r	Ames 29172	7.50L-d	Ames 18571	4.94e-n	Ames 29172	15.81Q-j	Ames 18571	12.01V-q	Ames 29172	61.51M-h	Ames 18571	7.19jkl
Ames 27870	54.30M-ı	Ames 18570	59.20G-b	Ames 27870	7.10N-f	Ames 18570	7.54L-d	Ames 27870	5.61l-q	Ames 18570	22.81I-S	Ames 27870	37.51V-l	Ames 18570	88.99H-T
Ames 27772	34.10m-r	Ames 18569	42.80c-q	Ames 27772	4.50j-n	Ames 18569	8.34G-V	Ames 27772	7.41g-q	Ames 18569	11.41Y-q	Ames 27772	31.51Z-l	Ames 18569	31.99Z-l
Ames 27771	36.66k-r	Ames 18568	71.20A-K	Ames 27771	6.62R-j	Ames 18568	10.34C-J	Ames 27771	12.57U-p	Ames 18568	34.41D-G	Ames 27771	20.19d-l	Ames 18568	123.59D-I
Ames 27770	30.36pqr	Ames 18567	43.80a-q	Ames 27770	7.62L-c	Ames 18567	5.34b-m	Ames 27770	8.37g-q	Ames 18567	5.21m-q	Ames 27770	29.79Z-l	Ames 18567	0.19lm
Ames 27392	46.16V-p	Ames 18566	62.20D-V	Ames 27392	7.62L-c	Ames 18566	8.14I-Y	Ames 27392	13.37S-o	Ames 18566	13.81R-o	Ames 27392	46.39Q-l	Ames 18566	61.39M-h
Ames 27391	50.96P-l	Ames 18565	50.80P-l	Ames 27391	7.22N-e	Ames 18565	6.74P-j	Ames 27391	19.97K-b	Ames 18565	11.81Y-q	Ames 27391	43.79R-l	Ames 18565	28.79Z-l
Ames 25170	49.96Q-m	Ames 18564	60.80E-Y	Ames 25170	7.02N-h	Ames 18564	7.94K-a	Ames 25170	19.37M-c	Ames 18564	12.61U-p	Ames 25170	53.79O-k	Ames 18564	44.39R-l
Ames 24926	59.16G-b	Ames 18563	60.60E-Z	Ames 24926	7.22N-e	Ames 18563	7.54L-d	Ames 24926	11.57Y-q	Ames 18563	16.41P-ı	Ames 24926	22.99c-l	Ames 18563	50.79P-k
Ames 24923	56.56I-f	Ames 18561	44.00a-q	Ames 24923	7.62L-c	Ames 18561	6.14U-l	Ames 24923	10.57b-q	Ames 18561	7.01ı-q	Ames 24923	27.79Z-l	Ames 18561	4.59kl
Ames 24921	52.43O-k	Ames 18560	49.20S-n	Ames 24921	6.75P-j	Ames 18560	7.54L-d	Ames 24921	10.51b-q	Ames 18560	11.41Y-q	Ames 24921	39.19U-l	Ames 18560	29.39Z-l
Ames 24917	54.56L-ı	Ames 18559	53.20N-j	Ames 24917	9.02E-Q	Ames 18559	8.34G-V	Ames 24917	20.17K-a	Ames 18559	20.81K-Y	Ames 24917	38.19V-l	Ames 18559	56.99N-ı
Ames 24909	59.96F-a	Ames 14363	25.50rs	Ames 24909	10.22C-K	Ames 14363	5.74Z-m	Ames 24909	40.57DE	Ames 14363	18.51N-e	Ames 24909	156.79CD	Ames 14363	22.69c-l
Ames 24907	70.96A-K	Ames 13900	70.00A-M	Ames 24907	9.82C-L	Ames 13900	12.74B	Ames 24907	22.97I-R	Ames 13900	72.68A	Ames 24907	98.79G-P	Ames 13900	380.19A
Ames 23642	55.76J-g	Ames 13899	60.00F-a	Ames 23642	11.42BCD	Ames 13899	11.14B-E	Ames 23642	32.17E-I	Ames 13899	62.61B	Ames 23642	130.19D-H	Ames 13899	200.19C
Ames 23641	44.16a-q	Ames 12778	33.10n-r	Ames 23641	5.82Y-m	Ames 12778	4.74g-n	Ames 23641	6.17k-q	Ames 12778	9.81d-q	Ames 23641	9.99ı-l	Ames 12778	0.19lm
Ames 23639	50.76P-l	Ames 10235	58.80G-c	Ames 23639	4.62ı-n	Ames 10235	10.14C-K	Ames 23639	4.77n-q	Ames 10235	43.61CD	Ames 23639	14.39g-l	Ames 10235	153.19CDE
Ames 23635	58.96G-c	Ames 10234	52.20O-l	Ames 23635	5.82Y-m	Ames 10234	8.74F-S	Ames 23635	8.37g-q	Ames 10234	30.41F-J	Ames 23635	20.99d-l	Ames 10234	73.19J-b

Differences between means indicated with the same letter are not significant. G/C: Genotypes/Cultivars, PH: Plant height, BN: Branch number, UM: Umbel number, UmbltN: Umbellet number

Table S3. 1000 fruit weight, fruit yield, biological yield and harvest index of different coriander genotypes.

G/C	1000FW	G/C	1000FW	G/C	FY	G/C	FY	G/C	BY	G/C	BY	G/C	HI	G/C	HI
PI 669966	6.12s-4	Ames 23634	11.69K-S	PI 669966	1.07P-e	Ames 23634	2.46H-c	PI 669966	3.59S-d	Ames 23634	13.11E-R	PI 669966	23.82b-p	Ames 23634	21.87f-r
PI 669965	6.07t-4	Ames 23632	14.16FGH	PI 669965	0.95P-e	Ames 23632	0.72U-e	PI 669965	4.34R-d	Ames 23632	1.36a-d	PI 669965	11.70t-y	Ames 23632	35.31E-S
PI 669964	14.06F-I	Ames 23627	7.73f-v	PI 669964	1.01P-e	Ames 23627	1.09P-e	PI 669964	5.87N-d	Ames 23627	3.44S-d	PI 669964	7.79vy	Ames 23627	26.14S-o
PI 669963	9.87P-e	Ames 23626	9.17T-k	PI 669963	0.79T-e	Ames 23626	0.93Q-e	PI 669963	3.07U-d	Ames 23626	2.63Y-d	PI 669963	13.38r-v	Ames 23626	35.89E-P
PI 669962	14.37EFG	Ames 23625	6.33r-4	PI 669962	1.43N-e	Ames 23625	1.15O-e	PI 669962	5.37P-d	Ames 23625	3.26S-d	PI 669962	20.75g-t	Ames 23625	35.71E-Q
PI 669961	13.72F-K	Ames 23624	5.83u-4	PI 669961	0.93Q-e	Ames 23624	2.11I-e	PI 669961	3.98R-d	Ames 23624	6.16N-d	PI 669961	13.10r-v	Ames 23624	34.67E-V
PI 669960	7.54h-z	Ames 23623	5.80u-4	PI 669960	0.83S-e	Ames 23623	2.03I-e	PI 669960	3.42S-d	Ames 23623	7.64J-d	PI 669960	12.43s-v	Ames 23623	24.12a-p
PI 669959	10.89L-U	Ames 23622	10.30N-a	PI 669959	1.49M-e	Ames 23622	1.39N-e	PI 669959	5.91N-d	Ames 23622	4.16R-d	PI 669959	19.15k-t	Ames 23622	32.26I-d
PI 664512	9.42T-h	Ames 23621	7.60g-y	PI 664512	3.09F-b	Ames 23621	1.99I-e	PI 664512	12.60F-T	Ames 23621	7.38J-d	PI 664512	20.37h-t	Ames 23621	24.52Z-p
PI 633685	10.54M-Z	Ames 23620	9.12T-l	PI 633685	4.85B-I	Ames 23620	1.31N-e	PI 633685	16.65C-K	Ames 23620	3.86R-d	PI 633685	25.88T-o	Ames 23620	33.06G-c
PI 531296	7.09k-2	Ames 23619	6.35r-4	PI 531296	1.29N-e	Ames 23619	1.39N-e	PI 531296	3.87R-d	Ames 23619	5.96N-d	PI 531296	30.59K-f	Ames 23619	17.44o-u
PI 531293	6.94m-2	Ames 23618	9.68S-g	PI 531293	2.67H-c	Ames 23618	1.69L-e	PI 531293	8.50I-d	Ames 23618	6.09N-d	PI 531293	28.17N-k	Ames 23618	24.56Y-p
PI 502320	6.84n-3	Ames 23616	6.73n-3	PI 502320	2.11I-e	Ames 23616	2.53H-c	PI 502320	8.72I-d	Ames 23616	10.13H-b	PI 502320	19.21j-t	Ames 23616	23.21d-p
PI 483232	10.87L-V	Ames 23614	16.38CDE	PI 483232	4.71B-J	Ames 23614	2.53H-c	PI 483232	12.70E-S	Ames 23614	6.62M-d	PI 483232	34.91E-U	Ames 23614	40.62B-J
PI 478378	8.29a-r	Ames 21655	6.95m-2	PI 478378	3.57C-U	Ames 21655	2.63H-c	PI 478378	8.22I-d	Ames 21655	9.40I-d	PI 478378	43.32B-E	Ames 21655	26.83P-n
PI 274290	15.24DEF	Ames 21108	8.80V-n	PI 274290	1.31N-e	Ames 21108	1.35N-e	PI 274290	3.64R-d	Ames 21108	3.60S-d	PI 274290	35.49E-R	Ames 21108	41.50B-I

PI 269472	7.82e-u	Ames 21105	10.35N-a	PI 269472	3.61C-T	Ames 21105	1.69L-e	PI 269472	11.21G-Z	Ames 21105	5.88N-d	PI 269472	37.21C-N	Ames 21105	25.85U-o
PI 269470	6.69o-3	Ames 20048	6.63o-3	PI 269470	1.91J-e	Ames 20048	3.35D-Y	PI 269470	6.65M-d	Ames 20048	15.29C-N	PI 269470	24.35Z-p	Ames 20048	20.76g-t
PI 268378	5.461-4	Ames 20046	6.40q-4	PI 268378	0.39a-e	Ames 20046	3.17F-b	PI 268378	1.88Z-d	Ames 20046	8.61I-d	PI 268378	29.67K-g	Ames 20046	38.37C-L
PI 256061	8.63Y-o	Ames 19089	6.50p-4	PI 256061	2.03I-e	Ames 19089	4.01B-N	PI 256061	5.78O-d	Ames 19089	10.00H-c	PI 256061	37.17C-N	Ames 19089	42.45B-F
PI 253146	5.38 234	Ames 18596	16.50CD	PI 253146	0.59Y-e	Ames 18596	1.57L-e	PI 253146	3.59S-d	Ames 18596	4.03R-d	PI 253146	21.23g-s	Ames 18596	37.60C-M
PI 249115	11.91J-P	Ames 18595	6.05t-4	PI 249115	0.95P-e	Ames 18595	4.33B-M	PI 249115	3.18T-d	Ames 18595	9.41I-d	PI 249115	34.46E-V	Ames 18595	46.27BC
PI 196843	9.91P-d	Ames 18594	9.45T-h	PI 196843	1.39N-e	Ames 18594	2.17I-e	PI 196843	4.34R-d	Ames 18594	6.58N-d	PI 196843	35.13E-T	Ames 18594	31.64J-e
PI 193770	14.03F-I	Ames 18592	6.10s-4	PI 193770	1.03P-e	Ames 18592	1.19N-e	PI 193770	3.04U-d	Ames 18592	2.94V-d	PI 193770	38.43C-L	Ames 18592	38.67C-K
PI 193769	15.11DEF	Ames 18591	6.37r-4	PI 193769	2.21I-e	Ames 18591	2.83H-c	PI 193769	26.21B	Ames 18591	7.18K-d	PI 193769	8.48uvy	Ames 18591	38.88C-K
PI 193493	11.68K-S	Ames 18590	8.95U-m	PI 193493	3.27D-Z	Ames 18590	3.05G-b	PI 193493	12.49F-U	Ames 18590	9.40I-d	PI 193493	26.91P-m	Ames 18590	31.59J-e
PI 174129	7.26j-2	Ames 18589	5.42 234	PI 174129	3.73B-R	Ames 18589	1.23N-e	PI 174129	11.93F-Y	Ames 18589	4.16R-d	PI 174129	31.94J-e	Ames 18589	26.31R-o
PI 172808	9.73R-f	Ames 18588	9.95O-d	PI 172808	5.17B-H	Ames 18588	2.11I-e	PI 172808	20.78B-F	Ames 18588	4.96Q-d	PI 172808	17.60n-u	Ames 18588	42.14B-G
PI 171592	8.58Y-p	Ames 18585	19.42AB	PI 171592	3.97B-O	Ames 18585	3.21E-a	PI 171592	16.07C-M	Ames 18585	8.73I-d	PI 171592	25.08Y-p	Ames 18585	41.66B-H
PI 170320	4.48 45	Ames 18587	7.37h-2	PI 170320	1.27N-e	Ames 18587	3.65C-S	PI 170320	5.96N-d	Ames 18587	10.50H-a	PI 170320	23.79c-p	Ames 18587	29.71K-g
PI 170319	7.93d-t	Ames 18583	6.20s-4	PI 170319	4.41B-L	Ames 18583	0.89R-e	PI 170319	17.61B-I	Ames 18583	3.63R-d	PI 170319	20.95g-t	Ames 18583	19.43j-t
Pelmus	7.29ı-2	Ames 18582	12.72G-L	Pelmus	1.41N-e	Ames 18582	2.11I-e	Pelmus	5.61O-d	Ames 18582	6.16N-d	Pelmus	25.42V-o	Ames 18582	32.93G-c
Gürbüz	10.33N-a	Ames 18581	8.47Z-q	Gürbüz	2.84H-c	Ames 18581	6.13BCD	Gürbüz	8.22I-d	Ames 18581	24.30BC	Gürbüz	34.39E-V	Ames 18581	24.89Y-p
Gamze	9.08T-l	Ames 18580	5.67v-4	Gamze	2.98H-b	Ames 18580	2.13I-e	Gamze	9.86I-c	Ames 18580	9.22I-d	Gamze	31.46J-e	Ames 18580	21.45f-s
Erbaa	7.54h-z	Ames 18578	4.80 345	Erbaa	3.78B-Q	Ames 18578	2.69H-c	Erbaa	13.87E-Q	Ames 18578	7.82J-d	Erbaa	26.56Q-o	Ames 18578	33.49F-Z
Arslan	17.37BC	Ames 18577	6.15s-4	Arslan	2.99G-b	Ames 18577	3.11F-b	Arslan	8.90I-d	Ames 18577	10.84H-a	Arslan	34.37E-V	Ames 18577	27.75O-l
Ames 4998	6.52p-4	Ames 18575	9.30T-j	Ames 4998	1.24N-e	Ames 18575	3.19E-b	Ames 4998	3.21T-d	Ames 18575	10.54H-a	Ames 4998	41.39B-I	Ames 18575	29.37L-h
Ames 23640	14.34E-H	Ames 18574	6.52p-4	Ames 23640	3.20E-a	Ames 18574	1.67L-e	Ames 23640	19.46B-H	Ames 18574	7.61J-d	Ames 23640	19.66ı-t	Ames 18574	19.70ı-t
Ames 29174	7.06l-2	Ames 18573	11.07L-T	Ames 29174	2.29I-d	Ames 18573	6.39BC	Ames 29174	15.05C-O	Ames 18573	24.34BC	Ames 29174	15.81p-v	Ames 18573	25.96T-o
Ames 29173	3.23 56	Ames 18572	20.74A	Ames 29173	0.69V-e	Ames 18572	0.76T-e	Ames 29173	4.64Q-d	Ames 18572	1.73a-d	Ames 29173	18.51l-t	Ames 18572	45.29BCD
Ames 29172	5.51z-4	Ames 18571	17.62BC	Ames 29172	1.83K-e	Ames 18571	1.36N-e	Ames 29172	6.91L-d	Ames 18571	3.45S-d	Ames 29172	28.35M-k	Ames 18571	41.79B-H
Ames 27870	12.61G-M	Ames 18570	8.09c-t	Ames 27870	1.93J-e	Ames 18570	3.80B-P	Ames 27870	8.57I-d	Ames 18570	14.56D-P	Ames 27870	23.99b-p	Ames 18570	28.07N-k
Ames 27772	11.71K-S	Ames 18569	21.49A	Ames 27772	0.83S-e	Ames 18569	2.56H-c	Ames 27772	2.79Y-d	Ames 18569	10.73H-a	Ames 27772	35.10E-U	Ames 18569	26.87P-n
Ames 27771	14.01F-I	Ames 18568	10.09O-c	Ames 27771	1.00P-e	Ames 18568	3.48D-V	Ames 27771	2.14Z-d	Ames 18568	8.77I-d	Ames 27771	35.83E-Q	Ames 18568	40.61B-J
Ames 27770	12.91G-L	Ames 18567	11.82K-Q	Ames 27770	1.16N-e	Ames 18567	0.02c-f	Ames 27770	3.38S-d	Ames 18567	0.83b-e	Ames 27770	31.60J-e	Ames 18567	32.84H-c
Ames 27392	10.21N-b	Ames 18566	9.27T-j	Ames 27392	1.72L-e	Ames 18566	2.72H-c	Ames 27392	6.49N-d	Ames 18566	12.37F-V	Ames 27392	27.80O-k	Ames 18566	24.85Y-p
Ames 27391	10.64M-Y	Ames 18565	8.17b-s	Ames 27391	5.84B-G	Ames 18565	0.80S-e	Ames 27391	4.56Q-d	Ames 18565	6.15N-d	Ames 27391	20.58g-t	Ames 18565	20.45g-t
Ames 25170	10.44N-Z	Ames 18564	6.52p-4	Ames 25170	1.44N-e	Ames 18564	1.42N-e	Ames 25170	7.32J-d	Ames 18564	8.02J-d	Ames 25170	22.84e-q	Ames 18564	22.78e-q
Ames 24926	7.54h-1	Ames 18563	5.52z-4	Ames 24926	0.34b-e	Ames 18563	1.04P-e	Ames 24926	1.54a-d	Ames 18563	4.71Q-d	Ames 24926	24.57Y-p	Ames 18563	28.71M-ı
Ames 24923	4.54 45	Ames 18561	15.09DEF	Ames 24923	0.34b-e	Ames 18561	0.62Y-e	Ames 24923	2.47Y-d	Ames 18561	2.57Y-d	Ames 24923	20.52g-t	Ames 18561	33.36F-a
Ames 24921	12.01I-O	Ames 18560	10.17O-c	Ames 24921	4.64B-K	Ames 18560	1.32N-e	Ames 24921	16.32C-L	Ames 18560	4.56Q-d	Ames 24921	48.52B	Ames 18560	33.82F-Y
Ames 24917	6.64o-3	Ames 18559	11.07L-T	Ames 24917	0.48Z-e	Ames 18559	3.14F-b	Ames 24917	8.54I-d	Ames 18559	8.07J-d	Ames 24917	11.95tuv	Ames 18559	40.08B-J
Ames 24909	11.76K-R	Ames 14363	1.34 67	Ames 24909	6.04B-E	Ames 14363	0.46def	Ames 24909	16.68C-J	Ames 14363	0.62cde	Ames 24909	36.89D-O	Ames 14363	16.03p-v
Ames 24907	7.96d-t	Ames 13900	5.54y-4	Ames 24907	2.02I-e	Ames 13900	9.58A	Ames 24907	6.38N-d	Ames 13900	50.78A	Ames 24907	31.47J-e	Ames 13900	19.44ı-t
Ames 23642	9.74Q-f	Ames 13899	6.02t-4	Ames 23642	0.72U-e	Ames 13899	5.92B-F	Ames 23642	6.49N-d	Ames 13899	23.86B-D	Ames 23642	73.36A	Ames 13899	26.02T-o
Ames 23641	16.89CD	Ames 12778	9.36T-ı	Ames 23641	1.04P-e	Ames 12778	0.6ef	Ames 23641	7.92J-d	Ames 12778	0.01de	Ames 23641	17.86m-t	Ames 12778	13.91q-v
Ames 23639	13.96F-J	Ames 10235	3.22 5-7	Ames 23639	1.46N-e	Ames 10235	6.04B-E	Ames 23639	6.11N-d	Ames 10235	22.12B-E	Ames 23639	25.92T-o	Ames 10235	28.44M-j

Ames 23635	17.06CD	Ames 10234	12.27H-N	Ames 23635	1.98J-e	Ames 10234	6.58B	Ames 23635	7.26J-d	Ames 10234	20.42B-G	Ames 23635	28.47M-j	Ames 10234	33.07G-b

Differences between means indicated with the same letter are not significant. G/C: Genotypes/Cultivars, 1000FW: 1000 fruit weight, FY: Fruit yield, BY: Biological yield, HI: Harvest index

Table S4. Minor essential oil compositions of the high-yielding coriander genotypes.

No	Essential oil compositions (%v/v)	RT (min)	Ames 10234	Ames 10235	Ames 13899	Gamze	Ames 13900	Ames 18559	Gürbüz	Ames 18566	Ames 18568	Ames 18569	Ames 18570	Ames 18573	Ames 18575	Ames 18577	Ames 18581	Ames 18587	Ames 18585	Ames 18590	Ames 18591	Ames 18595
1	α-Pinen	16.68	1.32	4.70	7.10	1.20	5.67	4.13	3.57	6.80	2.68	3.53	3.37	5.72	5.34	5.90	4.72	0.22	2.89	5.73	6.08	5,71
2	Camphene	17.44	0.23	0.64	2.88	0.23	1.89	0.95	0.99	2.20	0.40	0.85	0.58	0.89	0.88	2.77	1.56	0.05	0.53	3.05	2.13	2,32
3	Sabiene	18.36	0.04	1.36	1.47	0.13	1.11	0.38	0.63	1.38	0.22	0.54	0.33	0.42	*nd*	1.95	0.97	0.07	0.39	1.63	1.22	1,01
4	β-Mycrene	18.74	3.30	1.54	3.75	1.28	3.02	2.21	2.29	2.97	1.60	2.05	1.97	2.10	1.71	2.91	2.40	0.54	1.38	2.89	2.92	2,74
5	I-phellandrene	19.38	2.04	2.25	1.27	0.53	1.07	0.66	0.74	0.61	0.45	0.53	0.57	0.48	0.26	0.46	0.39	0.21	0.25	0.38	0.45	0,43
6	α-Terpinen	19.86	2.87	2.90	2.11	0.76	2.01	1.11	1.37	1.30	0.80	1.04	1.04	0.87	0.51	1.25	0.83	0.20	0.46	1.04	1.00	0,81
7	di-Limonen	20.21	3.77	1.17	3.26	2.46	2.97	3.09	2.53	3.25	2.36	2.83	2.68	2.16	1.55	3.46	3.27	1.89	2.29	3.01	4.04	3,88
8	Delta-3-carene	20.79	3.68	2.88	2.40	1.15	2.14	1.78	1.75	1.51	1.31	1.46	1.82	1.64	0.97	0.96	0.94	0.33	0.73	0.26	0.97	1,02
9	α-Terpinolene	22.2	3.07	2.79	3.77	1.41	3.45	2.72	3.06	2.78	1.45	2.54	1.77	1.53	1.49	3.61	3.00	0.51	1.49	3.45	3.65	3,85
10	Thoujanol	24.22	2.11	0.17	0.45	0.05	0.63	0.29	0.81	0.44	0.05	0.47	0.53	2.53	0.41	1.57	0.66	0.07	0.25	1.55	0.74	0,59
11	Citronella	25.42	0.07	0.51	0.37	0.19	0.43	0.28	0.57	0.40	0.19	0.38	0.22	0.42	0.50	0.81	0.50	0.16	0.15	0.49	0.70	0,37
12	Nerol methylether	26.04	5.79	0.54	1.56	0.24	1.79	1.97	1.74	1.93	0.11	1.70	0.02	0.10	0.02	6.27	1.79	0.01	0.05	1.35	1.99	2,26
13	4-Terpineol	26.27	0.65	0.41	1.25	0.26	1.77	0.73	1.77	1.19	1.01	1.21	1.38	0.87	0.93	1.93	1.43	0.48	0.67	1.54	1.58	1,11
14	1-borneol	26.53	4.93	1.09	1.24	0.55	1.84	1.76	5.66	2.32	4.89	0.89	2.35	1.93	4.27	2.56	5.15	0.36	0.71	3.37	1.73	2,32
15	Farnesol methylether	26.71	1.43	2.91	2.23	2.54	1.89	1.48	0.98	1.06	1.27	1.00	1.45	1.01	0.52	0.51	0.47	0.31	0.67	0.46	0.71	0,63
16	α-Terpineol	26.91	2.21	1.92	2.99	0.94	3.91	3.59	3.70	2.65	2.07	2.61	2.65	2.71	2.41	3.91	3.21	1.77	1.50	3.16	3.31	3,26
17	β-Citronellol	27.78	0.15	0.36	0.25	0.10	0.22	0.24	0.51	0.26	0.24	0.31	0.19	0.30	0.56	0.61	0.32	0.10	0.10	0.41	0.68	0,38
18	Nerol	27.92	0.27	0.40	0.35	0.17	0.39	0.35	0.53	0.27	0.23	0.39	0.48	0.39	0.39	0.38	0.31	0.18	0.17	0.36	0.43	0,38
19	Trans-Geraniol	28.61	4.72	4.40	4.85	2.70	5.26	4.62	5.08	4.72	8.46	3.86	5.50	4.87	4.66	4.00	4.11	3.38	6.72	3.94	4.47	4,37
20	Cis-Geraniol	28.66	*nd*	*nd*	*nd*	*nd*	*nd*	*nd*	1.13	*nd*	*nd*	1.15	*nd*	*nd*	*nd*	0.98	1.22	0.90	*nd*	0.81	1.39	1,38
21	2-Decenol	29.07	0.69	0.89	0.45	0.25	1.29	1.95	0.96	1.07	0.43	0.29	1.36	0.87	1.15	1.61	0.90	0.81	0.25	0.89	0.42	0,13
22	2-Citral	29.53	0.43	0.50	0.43	0.46	0.52	0.53	0.81	0.44	0.52	0.52	0.74	0.71	0.53	0.56	0.62	8.76	0.39	0.54	0.61	0,46
23	Undecanal	29.8	0.21	1.17	0.49	0.18	0.65	1.10	0.89	0.85	0.18	1.17	0.49	1.53	1.67	0.83	0.94	0.48	0.27	0.94	0.44	0,50
24	Myrtenyl acetate	30.37	0.33	0.21	1.58	0.36	1.03	1.71	0.84	0.81	0.57	0.29	0.69	0.51	0.76	1.64	1.16	0.18	0.30	1.10	1.47	1,23
25	Citronellyl acetate	30.64	0.26	0.38	0.14	0.15	0.17	0.26	0.09	0.09	0.12	0.07	0.17	0.32	0.47	0.27	0.10	0.09	0.12	0.20	0.30	0,12
26	Nerol acetate	30.99	0.26	0.42	0.49	0.31	0.49	0.67	0.31	0.32	0.38	0.33	0.33	0.60	0.64	0.70	0.38	0.09	0.29	0.43	0.58	0,38
27	Isothymol	31.22	0.11	0.04	0.12	0.15	0.33	0.35	0.35	0.14	0.35	0.10	0.29	0.18	0.07	0.50	0.33	2.04	0.11	0.30	0.33	0,18
28	Geranyl acetate	31.49	9.94	7.39	6.82	13.06	7.35	7.49	5.50	5.99	11.05	5.30	6.80	7.20	6.13	5.47	5.55	6.08	10.49	5.31	6.03	6,93
29	n-Dodecanal	32.56	0.31	1.10	0.40	0.26	0.60	1.56	1.11	1.14	0.56	1.30	1.09	1.85	1.40	0.83	1.27	0.49	0.52	1.07	1.66	0,64
30	Trans-Caryophyllene	32.74	0.96	0.45	1.64	0.99	1.34	1.34	0.77	0.84	0.39	0.24	0.74	1.22	0.05	1.67	1.04	0.59	0.57	1.35	1.43	1,08

31	Tetradecanal	34.25	0.01	1.09	0.06	0.04	0.17	0.49	0.93	0.78	0.17	4.10	0.10	2.75	3.23	0.27	1.16	*nd*	0.11	1.49	0.39	0,93
32	2-dodecenal	34.58	2.85	5.99	1.84	1.18	2.29	6.60	2.94	3.29	1.77	4.43	5.35	4.95	4.98	2.54	3.43	1.25	1.63	2.66	2.13	2,03
33	Oleic acid	36.36	0.11	0.17	0.35	1.91	0.23	0.80	2.49	0.71	0.31	0.18	0.38	0.34	0.60	0.26	0.16	1.66	0.13	0.12	1.66	1,23
34	Hexadecanal	37.59	0.17	1.03	0.31	0.71	0.43	1.14	0.62	0.54	0.23	0.85	0.58	1.39	1.17	0.50	0.77	0.18	0.25	0.80	0.61	0,59
35	Caryophyllene oxide	38.08	0.24	0.08	0.28	1.33	0.34	0.33	0.22	0.14	0.14	0.17	0.40	0.40	0.13	0.42	0.21	0.32	0.22	0.17	0.22	0,40
36	13-tetradecenal	39.58	0.81	7.70	2.55	0.75	3.54	3.52	4.66	4.58	0.44	6.69	3.77	6.25	7.06	2.99	5.17	0.65	1.56	4.72	3.50	6,05
37	Mystric acid	42.63	0.13	0.85	0.99	2.03	0.97	4.11	1.70	2.66	0.39	4.10	0.41	2.01	2.00	1.38	1.53	8.53	0.12	1.92	2.55	3,19
38	2-Pentadecenanone	44.39	0.30	0.52	1.10	0.33	0.33	0.61	0.34	0.27	0.43	0.68	0.15	1.25	0.36	0.29	1.92	0.40	0.25	0.69	0.84	0,40
Total			60.77	62.92	63.59	41.34	63.53	66.90	64.94	62.70	48.22	60.15	52.74	65.27	59.78	69.53	63.89	44.34	38.98	63.58	65.36	65.29

RT: Retention time, *nd*: Not detected

Table S4. Continue

Essential oil compositions (%v/v)	Ames 19089	Ames 20048	Ames 23635	Ames 23634	Ames 23640	Ames 24907	Ames 24909	Arslan	Ames 24921	Pelmus	Ames 29174	PI 171592	PI 174129	PI 193493	PI 269472	Erbaa	PI 478378	PI 483232	PI 633685	PI 664512
α-Pinen	4.69	4.59	1.00	4.71	3.69	2.84	6.73	5.40	2.26	5.23	*nd*	5.91	*nd*	6.35	3.27	1.83	6.35	6.75	5.11	5.12
Camphene	0.79	0.26	0.21	0.13	0.98	0.46	1.60	1.01	0.24	0.89	*nd*	2.03	*nd*	1.82	0.26	0.35	2.14	2.83	1.16	0.87
Sabiene	0.24	0.17	0.07	0.40	0.53	0.29	0.97	0.62	0.20	0.48	*nd*	0.90	*nd*	1.21	0.19	0.16	1.10	1.68	0.67	0.72
β-Mycrene	1.63	1.10	0.94	1.14	2.20	1.21	2.12	1.89	0.93	1.57	*nd*	2.92	*nd*	2.62	1.29	0.91	2.77	3.10	1.95	1.86
I-phellandrene	0.30	0.23	0.22	0.24	0.28	0.19	0.26	0.27	0.14	0.17	*nd*	0.40	*nd*	0.33	0.20	0.13	0.32	0.35	0.23	0.19
α-Terpinen	0.59	0.41	0.38	0.45	0.63	0.38	0.59	0.65	0.31	0.37	*nd*	0.76	*nd*	0.78	0.43	0.25	0.68	0.79	0.58	0.47
di-Limonen	2.41	0.68	1.47	0.71	4.45	2.49	3.39	2.78	0.97	2.86	0.59	3.53	1.63	3.57	1.11	1.60	3.91	4.17	2.93	3.16
Delta-3-carene	1.14	0.82	0.68	0.77	0.96	0.54	0.70	0.86	0.62	0.61	*nd*	*nd*	*nd*	0.94	0.96	0.52	0.85	0.58	0.85	0.61
α-Terpinolene	1.41	0.57	0.91	0.49	2.88	1.14	2.46	2.14	0.59	2.50	*nd*	*nd*	*nd*	2.90	0.61	1.01	3.38	3.91	2.47	1.95
Thoujanol	0.24	2.59	0.14	0.18	0.42	0.13	0.33	0.57	0.22	0.37	*nd*	*nd*	*nd*	*nd*	0.30	0.24	0.51	0.91	0.49	0.45
Citronella	0.32	0.21	1.45	0.38	0.33	0.23	0.38	0.31	0.31	0.20	*nd*	*nd*	*nd*	*nd*	0.40	0.38	0.36	0.54	0.71	0.28
Nerol methylether	0.04	0.17	0.08	0.07	0.03	0.07	0.02	2.35	0.04	0.73	*nd*	*nd*	*nd*	2.00	0.05	0.03	2.26	1.81	2.38	0.01
4-Terpineol	0.81	3.42	0.32	0.63	1.07	0.47	0.86	1.22	0.47	0.15	*nd*	*nd*	*nd*	1.15	0.71	0.56	1.01	1.64	0.2	0.93
1-borneol	3.23	0.08	7.91	0.76	5.98	0.40	0.68	4.02	2.58	2.94	*nd*	*nd*	*nd*	0.75	0.49	9.29	2.00	0.18	2.07	1.14
Farnesol methylether	0.68	0.59	0.08	0.62	0.57	0.55	0.04	0.47	0.41	0.49	*nd*	*nd*	*nd*	0.51	0.63	0.01	0.43	1.69	0.5	2.17
α-Terpineol	2.44	1.45	1.35	2.18	3.41	1.00	2.37	4.11	1.76	2.43	0.37	3.03	*nd*	2.66	1.19	2.87	2.93	0.30	3.14	1.85
Beta-Citronellol	0.15	0.86	0.23	0.10	0.14	0.12	0.36	0.35	0.21	0.22	*nd*	0.37	*nd*	0.24	0.19	0.30	0.20	3.46	0.63	0.15
Nerol	0.24	1.34	0.06	0.15	0.18	0.18	0.27	0.31	0.21	0.25	*nd*	0.36	*nd*	0.23	0.25	0.21	0.23	0.39	0.38	0.21
Trans-Geraniol	5.43	0.09	0.47	3.14	7.01	3.66	6.49	5.31	4.98	5.58	1.06	3.85	*nd*	4.02	4.68	7.66	5.30	5.32	4.1	5.73
Cis-Geraniol	*nd*	0.23	0.41	*nd*	*nd*	*nd*	*nd*	*nd*	*nd*	*nd*	0.15	1.35	18.36	1.60	*nd*	*nd*	*nd*	1.51	1.69	*nd*
2-Decenol	0.41	0.09	0.37	0.92	0.93	0.22	0.46	1.99	0.96	0.61	*nd*	0.84	*nd*	0.82	0.42	0.87	0.68	0.28	1.06	0.40
2-Citral	0.50	0.23	0.65	0.33	0.48	0.33	0.42	0.56	0.46	0.44	2.14	0.59	*nd*	0.79	0.41	0.62	0.47	0.58	0.67	0.60
Undecanal	1.04	0.40	0.35	0.81	0.67	0.17	0.72	1.20	0.50	0.32	2.14	0.39	*nd*	0.26	0.78	0.87	0.29	0.34	0.8	0.18
Myrtenyl acetate	0.51	1.27	0.25	0.74	0.52	0.06	0.13	0.66	0.41	0.72	*nd*	0.85	0.21	0.71	0.54	0.15	0.82	0.25	0.81	0.12

Citronellyl acetate	0.16	0.41	0.35	0.18	0.08	0.08	0.12	0.27	0.17	0.18	*nd*	0.25	*nd*	0.28	0.53	0.14	0.24	0.08	0.32	0.68
Nerol acetate	0.43	0.18	0.34	0.34	0.26	0.15	0.38	0.46	0.44	0.47	*nd*	0.44	*nd*	0.02	0.68	0.32	0.27	0.28	0.51	0.14
Isothymol	0.13	0.45	0.15	0.21	0.19	0.21	0.25	0.34	0.28	0.09	1.73	0.18	*nd*	0.27	0.26	0.15	7.30	0.29	0.31	0.36
Geranyl acetate	14.94	0.10	8.91	14.21	9.45	5.83	7.28	6.99	10.32	7.83	2.31	5.91	*nd*	6.59	14.14	9.93	0.61	6.04	7.48	0.24
n-Dodecanal	0.70	10.47	0.36	0.73	0.86	0.48	0.72	1.25	0.95	1.02	*nd*	0.72	*nd*	0.65	1.03	1.01	1.22	0.44	0.99	7.53
Trans-Caryophyllene	0.47	1.35	0.14	1.89	0.83	0.63	0.92	0.48	0.20	1.97	0.45	5.58	9.32	1.46	1.12	0.57	1.22	1.61	0.94	0.55
Tetradecanal	1.96	1.98	0.18	0.14	0.36	0.13	0.71	0.44	1.07	0.93	*nd*	0.26	*nd*	0.13	1.54	0.86	0.35	0.43	2.40	3.58
2-dodecenal	6.15	1.07	1.17	5.87	3.46	0.85	3.70	5.13	8.04	7.20	*nd*	3.41	*nd*	4.77	5.14	5.98	4.60	1.85	4.80	4.83
Oleic acid	0.14	5.01	0.29	0.41	0.23	0.12	0.25	0.53	0.44	0.43	*nd*	0.26	*nd*	0.32	1.77	0.40	0.29	1.41	0.49	0.65
Hexadecanal	0.70	0.05	0.13	0.37	0.46	0.27	0.53	0.75	0.53	0.57	*nd*	0.52	4.05	0.40	0.86	0.78	0.39	0.36	0.83	0.33
Caryophyllene oxide	0.10	1.09	0.13	0.23	0.14	0.08	0.25	0.12	0.14	0.10	*nd*	0.66	*nd*	3.00	0.28	0.16	0.30	0.29	0.37	2.38
13-tetradecenal	10.39	8.68	1.41	2.54	4.28	0.96	6.79	5.51	2.89	3.10	*nd*	2.27	0.64	1.37	8.09	6.79	1.64	2.34	5.42	1.28
Mystric acid	1.54	1.33	0.07	0.36	0.30	0.23	1.58	1.36	0.62	0.95	7.41	0.64	2.29	1.38	0.68	1.19	5.36	1.43	3.00	1.70
2-Pentadecenanone	0.55	0.71	0.31	0.28	0.30	0.34	0.45	1.66	0.72	0.98	3.15	0.93	18.34	0.85	0.50	0.65	0.80	0.45	0.73	1.39
Total	67.60	54.73	33.94	47.81	59.54	27.49	56.28	64.34	46.59	55.95	21.50	50.11	54.84	57.75	55.98	59.75	63.58	60.66	64.17	54.81

nd: Not detected

Table S5. Minor fixed oil acids of the different origin coriander genotypes and cultivars.

Fixed acids	RT (min)	Ames 10234	Ames 10235	Ames 13899	Ames 13900	Ames 18559	Ames 18566	Ames 18568	Ames 18569	Ames 18570	Ames 18573	Ames 18575	Ames 18577	Ames 18581	Ames 18587	Ames 18585	Ames 18590	Ames 18591	Ames 18595	Ames 19089	Ames 20048
myristic asit (C14:0)	13.44	*nd*	*nd*	*nd*	*nd*	*nd*	2.48	0.33	2.01	*nd*	2.81	2.94	2.61	2.65	4.48	1.04	1.30	1.10	0.51	0.84	2.98
pentadecanoic asit (C15:0)	15.33	*nd*	*nd*	0.21	0.14	*nd*	2.31	0.19	*nd*	*nd*	*nd*	*nd*	11.73	1.71	1.62	*nd*	*nd*	*nd*	*nd*	*nd*	*nd*
cis-10-pentadecanoic asit (C15:1)	16.95	*nd*	*nd*	*nd*	*nd*	0.81	*nd*	*nd*	*nd*	*nd*	*nd*	*nd*	*nd*	1.57	1.57	*nd*	*nd*	*nd*	*nd*	*nd*	*nd*
palmitoleic asit (C16:1)	18.39	*nd*	*nd*	*nd*	*nd*	1.45	*nd*	*nd*	*nd*	*nd*	*nd*	*nd*	*nd*	*nd*	*nd*	*nd*	*nd*	*nd*	*nd*	*nd*	*nd*
heptadecanoic asit (C17:0)	19.12	*nd*	*nd*	*nd*	*nd*	*nd*	*nd*	*nd*	*nd*	0.07	0.55	*nd*	*nd*	*nd*	*nd*	*nd*	*nd*	1.04	1.17	*nd*	*nd*
oleic asit (C18:1n9c)	22.29	*nd*	0.84	*nd*	*nd*	*nd*	*nd*	*nd*	*nd*	*nd*	*nd*	*nd*	*nd*	*nd*	*nd*	*nd*	*nd*	*nd*	*nd*	*nd*	*nd*
linoleic asit (C18:2n6c)	23.39	0.65	*nd*	*nd*	*nd*	*nd*	3.73	*nd*	1.13	1.99	2.84	5.85	3.44	3.12	3.41	2.82	2.02	*nd*	3.60	3.79	3.47
cis-11-eicosenoic asit (C20:1)	25.45	*nd*	*nd*	*nd*	*nd*	*nd*	*nd*	*nd*	*nd*	*nd*	1.50	*nd*	0.78	*nd*	*nd*	*nd*	1.58	1.03	*nd*	*nd*	1.99
linolenic asit (C18:3n6)	26.58	*nd*	*nd*	*nd*	*nd*	*nd*	*nd*	*nd*	*nd*	*nd*	1.98	3.76	3.57	5.69	4.87	1.70	*nd*	1.83	2.39	1.80	1.66
heneicosanoic asit (C21:0)	26.61	3.29	0.92	*nd*	*nd*	*nd*	4.20	*nd*	0.62	*nd*	0.03	*nd*	*nd*	*nd*	*nd*	*nd*	*nd*	*nd*	*nd*	*nd*	*nd*
cis-11,14-eicosadienoic asit (C20:2)	27.64	*nd*	*nd*	*nd*	*nd*	*nd*	2.88	*nd*	3.03	2.56	4.56	*nd*	3.06	1.65	1.08	*nd*	*nd*	*nd*	*nd*	5.28	6.19
arachidonic asit (C20:4n6)	29.04	8.29	*nd*	*nd*	*nd*	*nd*	11.67	*nd*	*nd*	1.70	*nd*	10.33	4.25	*nd*	*nd*	2.95	4.56	3.86	*nd*	3.74	5.40
lignoceric asit (C24:0)	30.37	*nd*	*nd*	*nd*	*nd*	*nd*	*nd*	*nd*	*nd*	*nd*	*nd*	*nd*	*nd*	*nd*	*nd*	*nd*	27.40	*nd*	*nd*	*nd*	*nd*
Total (%v/v)		12.23	1.76	0.21	0.14	2.26	27.27	0.52	6.79	6.32	14.27	22.88	29.44	16.39	17.03	8.51	36.86	8.86	7.67	15.45	21.69

RT: Retention time, nd: Not detected

Table S5. Continue

Fixed acids	RT (min)	Ames 23635	Ames 23634	Ames 23640	Ames 24907	Ames 24909	Ames 24921	Ames 29174	PI 171592	PI 174129	PI 193493	PI 269472	PI 478378	PI 483232	PI 633685	PI 664512	Gamze	Gürbüz	Arslan	Pelmus	Erbaa
myristic asit (C14:0)	13.44	2.05	1.03	0.99	*nd*	*nd*	1.53	3.10	1.00	2.13	1.66	2.45	2.31	*nd*	1.09		2.54	2.48		2.82	2.61
pentadecanoic asit (C15:0)	15.33	*nd*	*nd*	*nd*	*nd*	*nd*	*nd*	*nd*	*nd*	*nd*	*nd*	*nd*	*nd*	*nd*	*nd*	*nd*	*nd*	*nd*	*nd*	*nd*	*nd*
cis-10-pentadecanoic asit (C15:1)	16.95	*nd*	*nd*	*nd*	*nd*	*nd*	*nd*	*nd*	*nd*	*nd*	*nd*	*nd*	*nd*	*nd*	*nd*	*nd*	*nd*	*nd*	*nd*	*nd*	*nd*
palmitoleic asit (C16:1)	18.39	*nd*	*nd*	*nd*	*nd*	*nd*	*nd*	*nd*	*nd*	*nd*	*nd*	*nd*	*nd*	*nd*	*nd*	*nd*	1.55	0.08	*nd*	*nd*	*nd*
heptadecanoic asit (C17:0)	19.12	*nd*	*nd*	*nd*	*nd*	*nd*	*nd*	*nd*	*nd*	*nd*	*nd*	*nd*	*nd*	*nd*	*nd*	*nd*	0.96	*nd*	*nd*	*nd*	*nd*
oleic asit (C18:1n9c)	22.29	*nd*	*nd*	*nd*	*nd*	*nd*	*nd*	*nd*	*nd*	*nd*	*nd*	*nd*	*nd*	*nd*	*nd*	*nd*	*nd*	*nd*	*nd*	*nd*	*nd*
linoleic asit (C18:2n6c)	23.39	2.59	1.02	1.07	*nd*	1.21	3.09	*nd*	4.32	8.19	3.72	5.23	5.69	2.37	1.48	3.56	*nd*	*nd*	5.09	*nd*	4.02
cis-11-eicosenoic asit (C20:1)	25.45		0.21	*nd*	*nd*	*nd*	*nd*	*nd*	*nd*	*nd*	*nd*	*nd*	*nd*	*nd*	*nd*	*nd*	*nd*	*nd*	*nd*	*nd*	*nd*
linolenic asit (C18:3n6)	26.58	1.07	0.53	*nd*	*nd*	2.11	*nd*	2.90	2.61	1.48	1.69	2.56	1.48	3.34	1.47	2.85	*nd*	*nd*	*nd*	2.82	4.14
heneicosanoic asit (C21:0)	26.61	*nd*	*nd*	*nd*	*nd*	*nd*	*nd*	*nd*	*nd*	*nd*	*nd*	*nd*	*nd*	*nd*	*nd*	*nd*	*nd*	1.36	*nd*	*nd*	*nd*
cis-11,14-eicosadienoic asit (C20:2)	27.64	3.30	2.03	*nd*	*nd*	*nd*	*nd*	*nd*	*nd*	*nd*	1.59	*nd*	1.33	*nd*	*nd*	*nd*	*nd*	*nd*	*nd*	1.76	*nd*
arachidonic asit (C20:4n6)	29.04	2.61	*nd*	*nd*	*nd*	*nd*	*nd*	*nd*	*nd*	*nd*	*nd*	*nd*	*nd*	*nd*	*nd*	*nd*	*nd*	4.73	*nd*	*nd*	*nd*
lignoceric asit (C24:0)	30.37	*nd*	*nd*	*nd*	*nd*	*nd*	*nd*	*nd*	*nd*	*nd*	*nd*	*nd*	*nd*	*nd*	*nd*	*nd*	*nd*	*nd*	*nd*	*nd*	*nd*
Total (%v/v)		11.62	4.82	2.06	0.00	3.32	4.62	6.00	7.93	11.80	8.66	10.24	10.81	5.71	4.04	6.41	5.05	8.65	5.09	7.40	10.77

RT: Retention time, nd: Not detected

Table S6. UPOV criteria of different origin coriander genotypes and cultivars.

G/C	ACF	IACF	ACHS	CS	DFP	CF	NLBL	STLL	SFBL	DIML	FS	IBCF
PI 669966	9	3	1	1	3	1	2	3	1	5	2	3
PI 669965	9	3	1	1	5	3	1	5	1	7	2	5
PI 669964	9	3	3	2	7	3	2	3	1	5	2	5
PI 669963	9	3	3	1	3	1	1	3	1	5	2	5
PI 669962	9	3	3	1	5	5	1	3	1	5	2	5
PI 669961	9	3	3	1	7	3	1	3	1	5	2	3
PI 669960	9	3	3	1	7	3	1	5	1	3	2	5
PI 669959	9	3	5	1	5	1	1	3	1	3	2	3
PI 664512	9	5	3	2	5	5	2	5	1	3	2	7
PI 633685	9	3	7	3	5	1	2	7	1	3	2	7
PI 531296	9	3	5	2	5	5	2	7	1	5	2	5
PI 531293	9	3	5	2	5	3	1	5	1	7	2	5
PI 502320	9	3	5	3	5	3	2	5	1	5	2	7
PI 483232	9	3	7	2	7	5	1	5	1	5	2	7
PI 478378	9	3	7	1	5	1	1	5	1	7	2	7
PI 274290	9	3	1	1	7	3	1	3	1	3	2	3
PI 269472	9	3	3	2	7	5	2	5	2	7	2	7
PI 269470	9	3	3	1	5	1	1	5	1	5	2	5
PI 268378	9	3	3	2	7	5	1	7	1	3	2	5
PI 256061	9	3	5	1	5	1	2	5	1	3	2	7
PI 253146	9	5	3	2	5	3	2	5	1	5	2	3
PI 249115	9	5	3	1	3	1	2	3	1	5	2	5
PI 196843	9	5	3	1	5	3	2	7	1	5	2	7
PI 193770	9	3	3	1	7	1	2	3	1	5	2	7
PI 193769	9	3	3	2	5	3	1	5	2	7	2	7
PI 193493	9	3	3	2	7	5	2	5	1	5	2	5
PI 174129	9	5	7	2	5	3	2	3	1	5	2	7
PI 172808	9	3	3	2	5	1	1	5	1	5	2	7
PI 171592	9	3	5	2	5	1	1	3	1	5	2	3
PI 170320	9	3	7	2	5	3	1	5	1	3	2	5
PI 170319	9	3	3	3	5	5	2	7	1	7	2	7
Pelmus	1	3	5	3	5	1	2	7	1	7	2	7
Gürbüz	9	3	5	2	5	1	1	5	1	5	2	5
Gamze	9	3	5	3	5	1	1	5	2	7	2	7
Erbaa	9	5	5	2	5	1	1	7	2	5	2	7
Arslan	9	3	3	2	7	3	1	3	1	5	2	5
Ames 4998	9	3	3	3	5	3	1	5	1	7	2	7
Ames 29174	9	5	5	3	5	3	2	7	2	7	2	7
Ames 29173	9	5	5	3	7	5	2	7	2	3	2	7
Ames 29172	9	5	5	3	7	5	1	7	2	5	2	7
Ames 27870	9	3	3	2	5	1	1	5	1	7	2	5
Ames 27772	9	3	3	1	5	1	2	3	2	3	2	3
Ames 27771	9	3	3	1	7	1	2	3	1	5	2	7
Ames 27770	9	3	3	1	7	3	1	5	1	5	2	5
Ames 27392	9	3	5	2	5	5	2	5	1	3	2	5
Ames 27391	9	3	7	2	5	3	2	7	1	5	2	5
Ames 25170	9	5	7	3	5	5	2	7	1	7	2	5
Ames 24926	9	3	5	2	7	3	1	7	1	7	2	5
Ames 24923	9	3	5	2	7	5	2	3	1	3	2	5

Ames 24921	9	3	5	3	5	3	2	3	1	3	2	7
Ames 24917	9	3	5	3	7	3	2	7	2	3	2	7
Ames 24909	9	5	5	3	5	5	2	5	2	5	2	7
Ames 24907	9	3	3	3	5	1	1	7	2	3	2	7
Ames 23642	9	3	5	3	5	3	2	7	2	3	2	7
Ames 23641	9	3	3	1	5	3	2	3	2	3	2	3
Ames 23640	9	3	3	2	5	1	2	5	2	5	2	5
Ames 23639	9	3	5	2	7	3	2	5	1	5	2	3
Ames 23635	9	3	3	3	5	3	1	5	2	3	2	3
Ames 23634	9	3	3	3	7	5	1	7	2	5	2	5
Ames 23632	9	3	5	2	5	5	1	5	2	3	2	7
Ames 23627	9	7	3	1	5	3	1	5	1	5	2	5
Ames 23626	9	3	1	1	3	1	1	3	1	3	2	7
Ames 23625	9	3	1	1	5	3	1	3	1	3	2	5
Ames 23624	9	5	3	2	7	5	2	5	2	3	2	3
Ames 23623	9	5	3	2	5	1	2	7	2	5	2	5
Ames 23622	9	3	3	2	5	1	1	5	1	5	2	3
Ames 23621	9	5	1	2	5	1	1	7	1	3	2	3
Ames 23620	9	3	3	2	7	5	1	3	1	3	2	5
Ames 23619	9	3	3	1	5	3	2	3	1	5	2	5
Ames 23618	9	5	3	1	3	1	1	3	1	5	2	3
Ames 23616	9	3	7	2	7	5	2	3	1	5	2	5
Ames 23614	9	3	3	1	7	3	1	3	1	5	2	5
Ames 21655	9	5	3	2	5	1	2	7	1	7	2	5
Ames 21108	9	5	3	1	7	5	2	3	1	5	2	5
Ames 21105	9	5	3	1	7	3	1	3	1	3	2	3
Ames 20048	9	3	7	1	5	5	2	5	1	3	2	5
Ames 20046	9	5	5	2	7	3	2	5	1	7	2	7
Ames 19089	9	3	3	2	5	1	2	5	1	7	2	7
Ames 18596	9	3	3	2	3	3	1	5	1	3	2	5
Ames 18595	9	5	5	2	5	1	1	5	1	7	2	7
Ames 18594	9	5	5	3	5	3	1	7	2	7	2	5
Ames 18592	9	3	7	2	5	5	1	7	2	7	2	7
Ames 18591	9	3	5	3	5	1	2	7	1	3	2	7
Ames 18590	9	3	7	3	5	1	2	5	1	7	2	7
Ames 18589	9	3	7	2	5	3	1	5	1	5	2	5
Ames 18588	9	3	5	3	5	1	2	5	1	7	2	5
Ames 18587	9	3	7	2	5	3	2	7	2	7	2	7
Ames 18585	9	3	3	2	3	3	2	7	2	3	2	5
Ames 18583	9	5	7	3	5	1	1	7	1	5	2	7
Ames 18582	9	3	3	3	3	3	1	3	1	7	2	5
Ames 18581	9	3	5	3	5	1	2	7	1	5	2	7
Ames 18580	1	3	7	1	5	3	2	3	1	7	2	7
Ames 18578	9	3	7	1	7	5	2	3	1	5	2	7
Ames 18577	9	7	5	1	5	5	2	7	1	5	2	5
Ames 18575	1	3	5	1	5	3	2	5	1	3	2	5
Ames 18574	9	3	5	2	5	1	1	7	1	5	2	7
Ames 18573	9	3	5	2	5	1	2	7	1	5	2	5
Ames 18572	9	5	3	1	5	1	2	5	1	3	2	5
Ames 18571	9	5	3	2	5	3	1	3	1	3	2	3
Ames 18570	9	5	5	3	5	3	1	5	1	7	2	7

Ames 18569	9	3	1	2	5	1	1	5	1	3	2	5
Ames 18568	9	5	3	1	7	5	1	3	2	7	2	7
Ames 18567	9	3	3	3	5	3	2	7	2	3	2	5
Ames 18566	9	5	7	3	7	5	1	3	2	5	2	7
Ames 18565	9	3	5	3	5	3	1	7	1	7	2	5
Ames 18564	9	3	3	2	5	1	1	5	1	7	2	5
Ames 18563	1	3	3	3	5	1	1	5	1	5	2	7
Ames 18561	1	3	3	2	5	5	1	7	1	5	2	3
Ames 18560	9	5	3	1	5	3	2	7	1	5	2	7
Ames 18559	9	3	5	3	5	3	1	5	2	3	2	7
Ames 14363	1	3	1	1	5	5	1	3	1	3	2	5
Ames 13900	9	3	7	1	7	3	2	7	2	5	2	7
Ames 13899	9	3	5	3	5	5	2	3	2	7	2	5
Ames 12778	1	3	1	2	3	3	2	3	1	3	2	3
Ames 10235	9	5	3	3	5	3	1	5	1	3	2	5
Ames 10234	9	3	3	3	7	3	2	7	1	5	2	5

G/C: Genotype/cultivar, ACF: Anthocyanin coloration in the flower, IACF: Intensity of anthocyanin coloration in the flowers, ACHS: Anthocyanin coloration of hypocotyl in seedling, CS: Cotyledon shape, DFP: Density of foliage in plant, CF: Coloration in foliage, NLBL: Number of leaflets in basal leaf, STLL: Size of terminal leaflet in leaf, SFBL: Structure of feathering in basal leaf, DIML: Density of incisions on margin in leaflet, FS: Fruit shape, IBCF: Intensity of brown color in fruit
